# Correlation study of malignant lymphoma and breast Cancer in different gender European populations: mendelian randomization analysis

**DOI:** 10.1186/s12863-023-01162-1

**Published:** 2023-10-09

**Authors:** Xiong Chen, GuoHuang Hu

**Affiliations:** Department of General Surgery, Affiliated Changsha Hospital of Hunan Normal University, Changsha, 410000 China

**Keywords:** Breast cancer, Malignant lymphoma, Gender, Mendelian randomization, Hodgkin disease

## Abstract

**Background:**

Previous research has already indicated an elevated risk of breast cancer (BC) among survivors of malignant lymphoma, but the underlying reasons remain unknown. Our objective is to elucidate the causal relationship between malignant lymphoma and BC through Mendelian randomization (MR). Genome-wide association studies (GWAS) data from 181,125 Hodgkin lymphoma (HL) patients and 181,289 non-Hodgkin lymphoma (NHL) patients from the FinnGen Consortium were utilized as exposure. We selected single nucleotide polymorphisms (SNPs) strongly associated with the exposure as instrumental variables to investigate their relationship with BC in a cohort of 107,722 participants. Subsequently, we obtained data from the UK Biobank containing gender-stratified information on HL, NHL, and BC. We validated the findings from our analysis and explored the impact of gender. The Inverse-Variance Weighted (IVW) method served as the primary reference for the two-sample MR, accompanied by tests for heterogeneity and pleiotropy.

**Results:**

The analysis results from the FinnGen consortium indicate that there is no causal relationship between HL and NHL with BC. HL (OR = 1.01, 95% CI = 0.98–1.04, p = 0.29), NHL (OR = 1.01, 95% CI = 0.96–1.05, p = 0.64). When utilizing GWAS data from the UK Biobank that includes different gender cohorts, the lack of association between HL, NHL, and BC remains consistent. HL (OR = 1.08, 95% CI = 0.74–1.56, p = 0.69), HL-Female (OR = 0.84, 95% CI = 0.59–1.19, p = 0.33), NHL (OR = 0.89, 95% CI = 0.66–1.19, p = 0.44), and NHL-Female (OR = 0.81, 95% CI = 0.58–1.11, p = 0.18).

**Conclusions:**

The two-sample MR analysis indicates that there is no significant causal relationship between malignant lymphoma (HL and NHL) and BC. The association between malignant lymphoma and breast cancer requires further in-depth research and exploration.

**Supplementary Information:**

The online version contains supplementary material available at 10.1186/s12863-023-01162-1.

## Introduction

Breast cancer (BC) is a common malignant tumor among women, and its high incidence and mortality rates have drawn significant attention [1]. Especially in the advanced stages, BC with metastasis is extremely challenging to cure [2]. Risk factors for BC have been identified, including alcohol consumption, body mass index (BMI), physical activity, height, smoking, age at menarche, age at menopause, type 2 diabetes, and family history of breast cancer (Illnesses of mother and siblings) [3–5]. Furthermore, Hodgkin lymphoma (HL) survivors who have undergone radiation therapy are at a considerably higher risk of developing BC [6]. Previous research has indicated that radiation therapy can increase the risk of BC [7]. However, the relationship between HL and BC remains unclear, and there is a scarcity of studies in this area.

HL patients have a notably high risk of developing secondary malignancies, with secondary BC being particularly prevalent among females [8]. Numerous studies have indicated that female HL patients who have undergone radiation therapy are at an elevated risk of developing BC (Risk = 22.3% ,95% CI:4.1–40.5) [9–12]. This heightened risk could be attributed to factors such as radiation exposure, hormonal influences, and age [13–15]. In a recent study, it was found that male HL patients also face an increased risk of BC (OR = 1.6, 95% CI: 0.7–3.3) [16]. Additionally, Kang et al. pointed out a significant correlation between the development of BC and an increased risk of non-Hodgkin lymphoma (NHL) (OR = 1.64, 95% CI = 1.34-2.00) [17]. Due to confounding factors, potential biases, and other complexities, the relationship between HL and BC, as well as between NHL and BC, remains unclear. The impact of gender on causality has also yet to be elucidated.

Mendelian randomization (MR) is a recently emerging method for inferring causal relationships, falling under the category of randomized controlled trial research [18–20]. The basic principle involves using the influence of randomly distributed genotypes on phenotypes to infer the relationship between exposure and outcomes [21]. It overcomes biases introduced by confounding factors and utilizes genetic variations strongly associated with the exposure to explore causal relationships with outcomes [22]. Because an individual’s genotype is established during conception, there will be no reverse causal relationship. The implementation of MR must satisfy three conditions: (1) the variation is correlated with the exposure, (2) the variation does not affect the outcome through confounding factors, (3) the variation affects the outcome solely through the exposure, without a direct influence [23]. The core of the research design is to establish a unidirectional causal relationship analysis where genetic variation influences outcomes through exposure. However, MR may also suffer from horizontal pleiotropy, meaning genetic variation may affect outcomes through other factors or directly, which should be eliminated for in our analysis [24, 25].

In this study, we conducted a two-sample MR analysis using summary data from genome-wide association studies (GWAS) sourced from public databases. The objective was to comprehensively elucidate the causal relationship between malignant lymphoma and BC, while also exploring whether gender exerts an influence. To the best of our knowledge, research in this realm is scarce. This endeavor holds the potential to offer valuable insights for the prevention and screening of BC in HL/NHL patients.

## Original research

## Methods

### Obtaining GWAS data related to malignant lymphoma exposure

In this study, we included exposure factors related to HL and NHL. These data were sourced from publicly available datasets, thus without any ethical and copyright concerns. We conducted our search on the website https://gwas.mrcieu.ac.uk/ and selected two GWAS datasets for HL and NHL from the year 2021 as instrumental variables (IVs). These datasets were both from the FinnGen consortium (Supplementary Table S1). We selected instrumental variables related to the exposure (p-value < 5 × 10 − 8), and the results indicated that the number of obtainable SNPs was quite limited. In such a scenario, conducting MR analysis could lead to issues of low statistical power and weak instrument problems, resulting in biased parameter estimates [26]. Therefore, we used a more liberal criterion of “p-value < 5 × 10 − 6” to identify SNPs significantly associated with the exposure while also addressing the elimination of linkage disequilibrium effects (LD r2 < 0.001, kb = 10,000). Additionally, we calculated the F-statistic for each SNP. To enhance the accuracy of our analysis, we applied criterion “F > 10” to filter out weak SNPs [27].

### GWAS data for BC and BC risk factors

The BC data included both male and female European populations and was sourced from the FinnGen consortium, comprising 8,401 cases and 99,321 controls. Additionally, to meet the conditions for Mendelian randomization implementation, we selected ten known risk factors for BC: Alcohol consumption, BMI, Physical activity, Height, Smoking, Age when periods started (menarche), Age at menopause, Type 2 diabetes (exclude DM1), and Family history of breast cancer (Illnesses of mother and siblings). The genetic association between risk factors and outcomes can be seen in Supplementary Figure S13. These data were obtained from UK Biobank, Within family GWAS consortium, MRC-IEU, and FinnGen consortiums. We utilized the inverse variance weighted (IVW) method to calculate the associations between HL/NHL and each of these BC risk factors. All the data were retrieved from https://gwas.mrcieu.ac.uk/ and are detailed in Supplementary Table S1.

### The data on HL/NHL and BC in different genders

In order to investigate whether the relationship between HL/NHL and BC is influenced by gender, and to validate the results of the analysis from the FinnGen consortium, we obtained data on HL/NHL and BC from the UK Biobank. The data was divided into two categories: one with both males and females, and the other with only females. The filtering criteria for SNPs were as follows: p-value < 5 × 10 − 6, LD r2 < 0.001, kb = 10,000, and F > 10. The detailed sources of the data refer to Supplementary Table S2.

### Statistical

We extracted the filtered SNPs from the exposure, removing those that were associated with the outcomes (p-value > 5 × 10 − 6). Next, the allelic directions of SNPs associated with exposure and outcomes were coordinated, while removing incompatible SNPs. For the two-sample MR analysis of HL/NHL and BC, we employed three methods: IVW, MR-Egger, and weighted median. The IVW estimates served as the primary reference for the MR analysis, while MR-Egger and weighted median were used as supplementary approaches due to their broader applicability despite lower efficiency. To assess the sensitivity of the analysis results, we conducted a heterogeneity test using Cochran’s Q estimate for the IVW method (Q-pvalue < 0.05 indicates potential heterogeneity) [28]. MR-Egger was utilized for directional pleiotropy testing, where pleiotropy-pval < 0.05 indicates the presence of directional pleiotropy [29]. Additionally, we employed the MR-PRESSO method to detect MR pleiotropic residuals, outliers, and correct for potential horizontal pleiotropy and outliers [25]. We cross-validated these two horizontal pleiotropy tests to provide more robust results or correct for any pleiotropy. Finally, we performed a leave-one-out analysis to examine whether individual SNPs introduced biases in the MR results.

Due to potential biases arising from discrepancies in results from different databases, we conducted cross-validation by utilizing two independent databases. Additionally, we explored the relationship between MR results and gender. The filtering criteria and MR methods used were consistent for both analyses. The aforementioned steps were carried out using the R-4.2.2 software packages TwoSampleMR, MRPRESSO, MungeSumstats, data.table, dplyr, and tidyr [30].

## Results

### Relationship between Hodgkin lymphoma and breast cancer

**Fig. 1:**
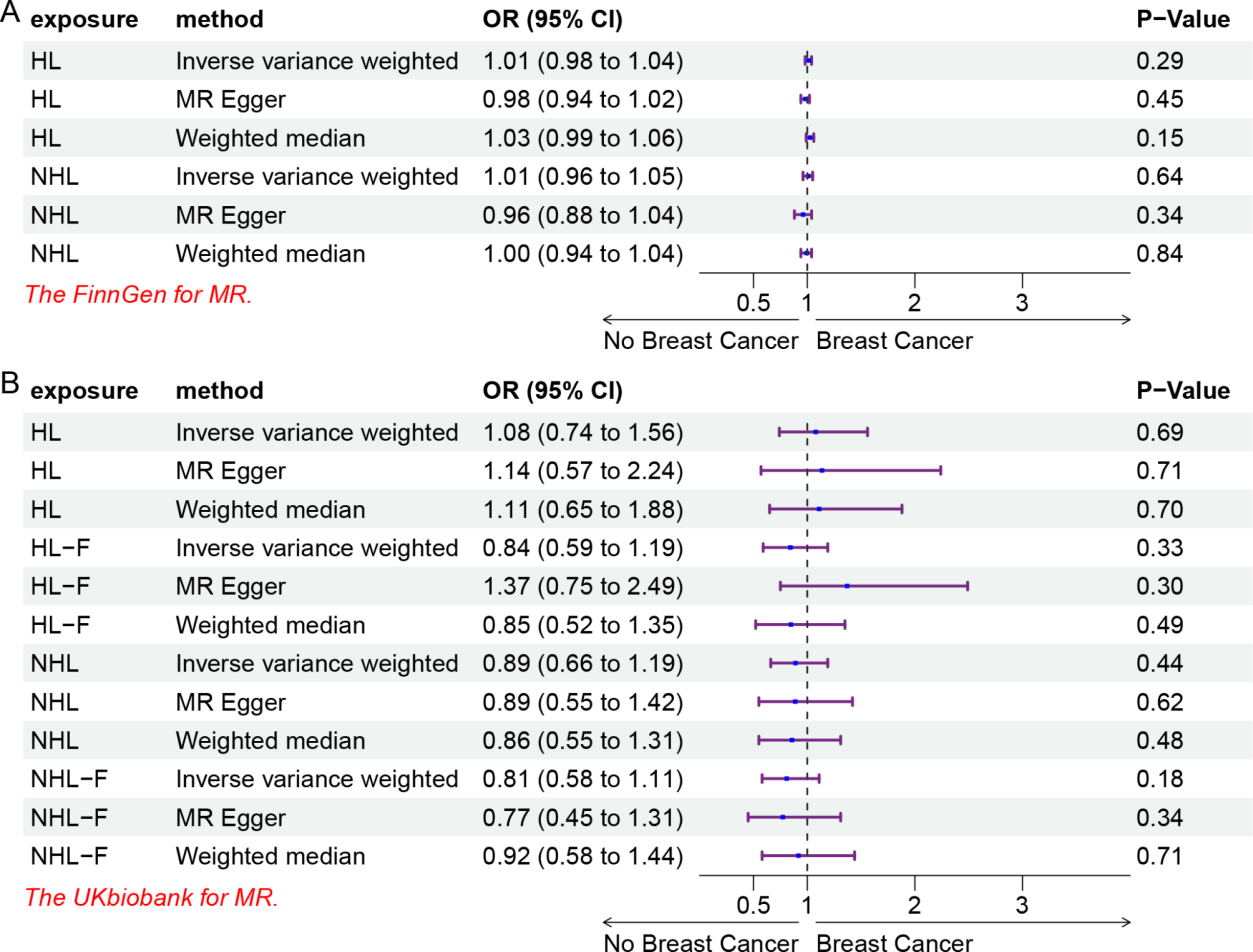
The forest plot for MR analysis: (A) MR analysis of HL, NHL, and BC data from the FinnGen consortium. (B) HL, NHL, BC data from different gender classifications of UK Biobank. HL: Hodgkin lymphoma; NHL: non Hodgkin lymphoma; HL-F: Hodgkin lymphoma-female; NHL-F: non Hodgkin lymphoma-female

We conducted an analysis of the GWAS data from the FinnGen consortium. We did not find a significant causal relationship between HL and BC (OR = 1.01, 95% CI = 0.98–1.04, p = 0.29). The results from MR-Egger (OR = 0.98, 95% CI = 0.94–1.02, p = 0.45) and Weighted Median (OR = 1.03, 95% CI = 0.99–1.06, p = 0.15) were consistent with the IVW method, all showing no significant association (Fig. 1A). The Cochran Q-test for IVW yielded a p-value of 0.515, suggesting no heterogeneity. MR-PRESSO did not detect any pleiotropy (p = 0.541), and there was no evidence of intercept presence (p = 0.117) (Table 1). Additionally, the leave-one-out analysis did not reveal any single SNP significantly altering the results (Supplementary Figure S1). The funnel plot was symmetrical (Supplementary Figure S7).

**Table 1:** Heterogeneity and pleiotropy tests in the two-sample MR analysis

Exposure	Heterogeneity-pval	Pleiotropy-pval	Presso-pval
HL finn	0.515	0.117	0.541
NHL finn	0.057	0.184	0.071
HL ukb	0.629	0.856	0.099
HL-F ukb	0.991	0.056	0.896
NHL ukb	0.569	0.986	0.704
NHL-F ukb	0.794	0.833	0.752

### Association between non-hodgkin lymphoma and breast cancer

We also investigated the potential causal relationship between NHL and BC, and similarly, no significant association was observed (OR = 1.01, 95% CI = 0.96–1.05, p = 0.64). Both MR-Egger and Weighted Median methods yielded non-significant p-values of 0.34 and 0.84, respectively (Fig. 1A). In sensitivity analysis, no heterogeneity was found (p = 0.057), and neither horizontal pleiotropy nor directional pleiotropy was detected (Pleiotropy-p = 0.184, Presso-p = 0.071) (Table 1). Additionally, the leave-one-out analysis and the funnel plot demonstrated the reliability of our findings, with no apparent bias (Supplementary Figure S2, 8).

### Association of HL/NHL with BC risk factors

**Fig. 2:**
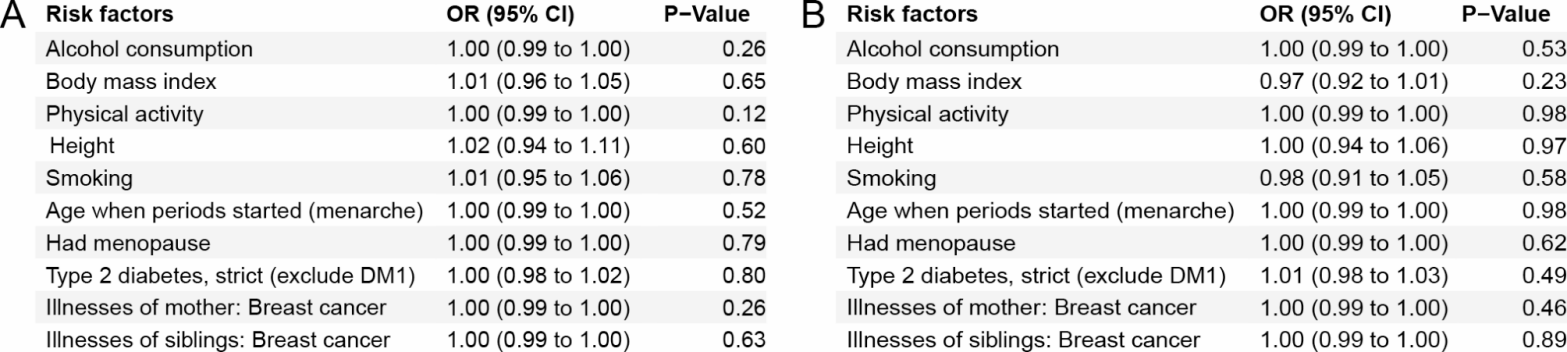
Association between malignant lymphoma and risk factors for BC: (A) Causal relationship between HL and BC risk factors. (B) Association of NHL with BC risk factors

To meet the requirements for MR analysis, it is crucial to establish a clear relationship between the exposure and outcome risk factors. Therefore, we selected ten established risk factors: Alcohol consumption, BMI, Physical activity, Height, Smoking, Menarche, Menopause, Type 2 diabetes, and Family history of breast cancer (Illnesses of mother and siblings). We conducted MR assessment using the IVW method. The results indicated that there was no causal relationship between HL and any of the eight risk factors (p > 0.05) (Fig. 2A). Similarly, the same observation was made for NHL (Fig. 2B).

### The causal effect from HL/NHL to BC in different genders

In order to explore whether there is a causal relationship between HL/NHL and BC patients of different genders and to enhance the credibility of our analysis. we performed two-sample Mendelian randomization separately for both genders: both males and females, and only females. The analysis of HL and BC in the combined gender group showed results similar to the data from the FinnGen consortium (OR = 1.08, 95% CI = 0.74–1.56, p = 0.69), indicating no significant causal association. The same observation was made for NHL as well (OR = 0.89, 95% CI = 0.66–1.19, p = 0.44). Interestingly, when analyzing only females with HL (HL-F) and only females with NHL (NHL-F), the results were also not associated significantly with BC (HL-F OR = 0.84, 95% CI = 0.59–1.19, p = 0.33; NHL-F OR = 0.81, 95% CI = 0.58–1.11, p = 0.18) (Fig. 1B). Furthermore, sensitivity tests did not detect any heterogeneity or pleiotropy issues (p > 0.05) (Table 1). The leave-one-out analysis and funnel plot were employed to demonstrate that the SNPs used for analysis exhibited neither singular bias nor uneven distribution (Supplementary Figure S3-6, S9-12).

## Discussion

Our research is the first to use a two-sample MR analysis to explore the causal relationship between Hodgkin lymphoma (HL), non-Hodgkin lymphoma (NHL), and breast cancer (BC). When utilizing gender-neutral GWAS data from the FinnGen consortium, the causal relationship was not found to be significant. This does not support the hypothesis that HL or NHL could increase the risk of BC. Interestingly, we conducted our analysis using gender-stratified GWAS data from the UK Biobank. HL and NHL were further divided into categories that included both males and females, as well as a category specific to females only. The analysis demonstrated that even when considering different gender scenarios, both HL and NHL are not significantly associated with an increased risk of BC.

The relationship between HL and BC has been extensively studied, while research on the association between NHL and BC remains relatively limited. Among survivors of HL, secondary cancers are a major cause of mortality, with BC being the most common in females [31]. Notably, women who have undergone treatment for HL exhibit a significantly elevated risk of developing BC [32]. However, the exact nature of the relationship between HL and BC remains unclear. Some scholars have proposed that factors such as the age at initial HL treatment [33], dosage and location of radiation [13], and hormonal stimulation may contribute to the occurrence of BC [14]. Nonetheless, most previous studies have utilized case-control or prospective research designs, which might introduce temporal ambiguity and reverse causation issues. Additionally, inevitable confounding factors and potential biases could be present. The underlying causes of secondary BC in HL patients might be related to other undiagnosed cancers or conditions. In contrast, our study employs MR analysis to explore the effects on outcomes based on exposures determined by genetic variations. This approach allows for the elimination of confounding factors and reverse causation interference, effectively elucidating causal relationships.

A recent study has revealed an evident increase in BC risk among male HL survivors [16]. This suggests that the causal relationship between HL and BC might be influenced by gender. However, due to the lower incidence of male BC and the resulting scarcity of cases, relevant research in this area is exceedingly limited. The data accessible from the UK Biobank is also relatively sparse, lacking GWAS data exclusively for males. Therefore, we conducted MR analysis using data categorized by both all genders and only females, aiming to explore the influence of gender on the relationship between HL and BC. The analysis results indicated no significant causal link between HL and BC in either of the two gender categories. It’s possible that the limitations of our data and the insufficient number of cases contributed to false negatives. This is an aspect that we and future related studies should seek to refine.

A history of prior malignant lymphoma is considered a negative prognostic factor for later developing BC [34]. For survivors of NHL, the challenge of subsequent late-stage complications is equally serious. A study focusing on the Korean population highlighted a significant increase in the risk of developing NHL for BC patients, particularly among younger BC patients who had undergone hormone therapy [17]. Additionally, lv et al. proposed that radiation treatment is a risk factor for secondary BC in female NHL patients [35]. This observation closely parallels findings from previous research on the relationship between HL and BC. However, because both of these studies are retrospective cohort studies, they could be influenced by confounding factors or potentially exhibit reverse causation. We hold reservations regarding the causal relationship between BC and NHL. As a next step, we devised a MR analysis for NHL and BC, also exploring the potential impact of gender on the causal relationship. While false negatives stemming from data limitations are a possibility, our results consistently demonstrate the absence of a causal relationship between NHL and BC.

After receiving improved treatment, both HL and NHL have achieved significant survival rates [36]. However, the increased risk of developing subsequent malignancies is a concern. Scholars generally believe that the treatment for HL might elevate the risk of developing a second type of cancer [37–39]. Some studies have observed a lower risk of BC among NHL survivors [40, 41], attributed to the potential ovarian suppression caused by NHL medications [42]. Similar circumstances are evident in HL cases. HL females undergoing chemotherapy experience a significant reduction in the incidence of BC due to ovarian suppression [34]. Nevertheless, the connection between malignant lymphoma and BC remains unclear. Our research aims to interpret the relationship between HL/NHL and BC from the perspective of genetic determinants influencing outcomes. This approach effectively mitigates confounding factors and offers an advantage over conventional epidemiological study methods. While our analytical results suggest there is no causal relationship between HL/NHL and BC, it is plausible that HL/NHL could impact the progression and prognosis of BC. BC patients with a history of malignant lymphoma exhibit poorer five-year disease-free survival (DFS) and overall survival (OS) [34]. Furthermore, this provides a reference point for exploring the interplay between malignant lymphoma and BC. As HL/NHL are not direct causes of BC, it implies that factors such as the treatments received, environment, lifestyle habits, and intrinsic physiological conditions could be associated with an increased risk of BC. Other variables, including certain unknown biological traits, require further investigation.

This study has three main strengths. Firstly, we employed a two-sample MR analysis. This approach utilizes allele randomization to determine biological phenotypes, followed by investigating the causal relationship between exposure phenotype and outcome using SNPs. This methodology effectively mitigates confounding factors and reverse causation. Secondly, MR analysis resembles a randomized controlled trial design. We conducted the analysis using publicly available GWAS data, making the process convenient and efficient. Thirdly, unlike previous studies, we simultaneously compared the relationship between both HL and NHL with breast cancer. Additionally, we explored whether gender has an impact on the results, a facet that hasn’t been addressed in prior research. However, our study also has several limitations. Firstly, the data available to us is limited. For instance, we couldn’t access data specific to only males, and there were constraints on the number of samples in the cohort. Secondly, The stratification of the samples was not done according to age, the other factors such as environmental and epigenetic factor are further need to be explored. Thirdly, further investigation is needed to determine if the findings of this MR study, which is conducted in individuals of European ancestry, can be generalized to non-European populations.

In conclusion, this study has found no significant causal relationship between HL/NHL and BC. This suggests that intensifying breast cancer screening and detection for malignant lymphoma patients might not yield effective results in clinical practice. Further exploration could potentially focus on treatment choices, environmental changes, and other unknown biological characteristics. More relevant research investigations are needed in the future to delve deeper into this matter.

### Electronic supplementary material

Below is the link to the electronic supplementary material.

Supplementary Material 1

## Data Availability

All the data used in this study are retrievable from public data platforms. For specific information, please refer to the supplementary materials.

## References

[1] DeSantis C, Siegel R, Bandi P, Jemal A (2011). Breast cancer statistics, 2011. CA Cancer J Clin.

[2] Harbeck N, Penault-Llorca F, Cortes J (2019). Breast cancer. Nat Rev Dis Primers.

[3] Singletary KW, Gapstur SM (2001). Alcohol and breast cancer: review of epidemiologic and experimental evidence and potential mechanisms. JAMA.

[4] Feigelson HS, Bodelon C, Powers JD (2021). Body Mass Index and Risk of Second Cancer among women with breast Cancer. J Natl Cancer Inst.

[5] Escala-Garcia M, Morra A, Canisius S (2020). Breast cancer risk factors and their effects on survival: a mendelian randomisation study. BMC Med.

[6] Opstal-van Winden AWJ, de Haan HG, Hauptmann M (2019). Genetic susceptibility to radiation-induced breast cancer after Hodgkin lymphoma. Blood.

[7] Ma YP, van Leeuwen FE, Cooke R (2012). FGFR2 genotype and risk of radiation-associated breast cancer in Hodgkin lymphoma. Blood.

[8] Bhatia S, Robison LL, Oberlin O (1996). Breast cancer and other second neoplasms after childhood Hodgkin’s disease. N Engl J Med.

[9] Moskowitz CS, Chou JF, Wolden SL (2014). Breast cancer after chest radiation therapy for childhood cancer. J Clin Oncol.

[10] Swerdlow AJ, Cooke R, Bates A (2012). Breast cancer risk after supradiaphragmatic radiotherapy for Hodgkin’s lymphoma in England and Wales: a National Cohort Study. J Clin Oncol.

[11] Aisenberg AC, Finkelstein DM, Doppke KP (1997). High risk of breast carcinoma after irradiation of young women with Hodgkin’s disease. Cancer.

[12] Schaapveld M, Aleman BMP, van Eggermond AM (2015). Second Cancer Risk up to 40 years after treatment for Hodgkin’s lymphoma. N Engl J Med.

[13] Roberti S, van Leeuwen FE, Ronckers CM (2022). Radiotherapy-Related dose and irradiated volume Effects on breast Cancer risk among Hodgkin Lymphoma Survivors. J Natl Cancer Inst.

[14] Travis LB, Hill DA, Dores GM (2003). Breast cancer following radiotherapy and chemotherapy among young women with Hodgkin disease. JAMA.

[15] Crump M, Hodgson D (2009). Secondary breast cancer in Hodgkin’s lymphoma survivors. J Clin Oncol.

[16] de Vries S, Krul I, Schaapveld M, et al. Risk of male breast cancer after Hodgkin lymphoma. Blood Blood. 2023;2023020940. 10.1182/blood.2023020940. 10.1182/blood.2023020940 37390297

[17] Kang D, Yoon SE, Shin D (2021). Risk of non-hodgkin lymphoma in breast cancer survivors: a nationwide cohort study. Blood Cancer J.

[18] Zoccali C, Testa A, Spoto B (2006). Mendelian randomization: a new approach to studying epidemiology in ESRD. Am J Kidney Dis.

[19] Smith GD, Ebrahim S (2003). Mendelian randomization: can genetic epidemiology contribute to understanding environmental determinants of disease?. Int J Epidemiol.

[20] Bowden J, Holmes MV (2019). Meta-analysis and mendelian randomization: a review. Res Synth Methods.

[21] Davies NM, Holmes MV, Davey Smith G (2018). Reading mendelian randomisation studies: a guide, glossary, and checklist for clinicians. BMJ.

[22] Sekula P, Del Greco MF, Pattaro C, Köttgen A (2016). Mendelian randomization as an Approach to assess causality using Observational Data. J Am Soc Nephrol.

[23] Boef AGC, Dekkers OM, le Cessie S (2015). Mendelian randomization studies: a review of the approaches used and the quality of reporting. Int J Epidemiol.

[24] Carter AR, Sanderson E, Hammerton G (2021). Mendelian randomisation for mediation analysis: current methods and challenges for implementation. Eur J Epidemiol.

[25] Verbanck M, Chen C-Y, Neale B, Do R (2018). Detection of widespread horizontal pleiotropy in causal relationships inferred from mendelian randomization between complex traits and diseases. Nat Genet.

[26] Wootton RE, Lawn RB, Millard LAC (2018). Evaluation of the causal effects between subjective wellbeing and cardiometabolic health: mendelian randomisation study. BMJ.

[27] Pierce BL, Ahsan H, VanderWeele TJ (2011). Power and instrument strength requirements for mendelian randomization studies using multiple genetic variants. Int J Epidemiol.

[28] Higgins JPT, Thompson SG, Deeks JJ, Altman DG (2003). Measuring inconsistency in meta-analyses. BMJ.

[29] Bowden J, Davey Smith G, Burgess S (2015). Mendelian randomization with invalid instruments: effect estimation and bias detection through Egger regression. Int J Epidemiol.

[30] Murphy AE, Schilder BM, Skene NG (2021). MungeSumstats: a Bioconductor package for the standardization and quality control of many GWAS summary statistics. Bioinformatics.

[31] Aleman BMP, van den Belt-Dusebout AW, Klokman WJ (2003). Long-term cause-specific mortality of patients treated for Hodgkin’s disease. J Clin Oncol.

[32] Travis LB, Hill D, Dores GM (2005). Cumulative absolute breast cancer risk for young women treated for Hodgkin lymphoma. J Natl Cancer Inst.

[33] Dores GM, Metayer C, Curtis RE (2002). Second malignant neoplasms among long-term survivors of Hodgkin’s disease: a population-based evaluation over 25 years. J Clin Oncol.

[34] Sanna G, Lorizzo K, Rotmensz N (2007). Breast cancer in Hodgkin’s disease and non-hodgkin’s lymphoma survivors. Ann Oncol.

[35] Lv X, Yue P, Zhou F (2023). Risk and prognosis of secondary breast cancer after radiation therapy for non-hodgkin lymphoma: a massive population-based analysis. Clin Transl Oncol.

[36] Henry-Amar M, Joly F (1996). Late complications after Hodgkin’s disease. Ann Oncol 7 Suppl.

[37] De Bruin ML, Burgers JA, Baas P (2009). Malignant mesothelioma after radiation treatment for Hodgkin lymphoma. Blood.

[38] Dores GM, Curtis RE, van Leeuwen FE (2014). Pancreatic cancer risk after treatment of Hodgkin lymphoma. Ann Oncol.

[39] van Eggermond AM, Schaapveld M, Lugtenburg PJ (2014). Risk of multiple primary malignancies following treatment of Hodgkin lymphoma. Blood.

[40] Travis LB, Curtis RE, Boice JD (1991). Second cancers following non-hodgkin’s lymphoma. Cancer.

[41] Brennan P, Coates M, Armstrong B (2000). Second primary neoplasms following non-hodgkin’s lymphoma in New South Wales, Australia. Br J Cancer.

[42] Mudie NY, Swerdlow AJ, Higgins CD (2006). Risk of second malignancy after non-hodgkin’s lymphoma: a british Cohort Study. J Clin Oncol.

