# Supplementary material for: Correlation study of malignant lymphoma and breast Cancer in different gender European populations: mendelian randomization analysis
Source: BMC Genom Data. 2023 Oct 9;24:59. doi: 10.1186/s12863-023-01162-1 (PMC10561426; doi:10.1186/s12863-023-01162-1)
Supplement: Supplementary file 1 — Supplementary Material 1 [file 12863_2023_1162_MOESM1_ESM.docx]

**Supplementary Table S1** Detailed information on GWAS data for Hodgkin lymphoma, non-Hodgkin lymphoma, breast cancer and breast cancer risk factors obtained from MRCIEU.

| **Phenotype** | **Year** | **Participants** | **Source** |
| --- | --- | --- | --- |
| Hodgkin lymphoma | 2021 | 181125 | [https://gwas.mrcieu.ac.uk/datasets/finn-b-CD2_HODGKIN_LYMPHOMA_EXALLC/](https://gwas.mrcieu.ac.uk/datasets/finn-b-CD2_HODGKIN_LYMPHOMA_EXALLC/) |
| Non Hodgkin lymphoma | 2021 | 181289 | [https://gwas.mrcieu.ac.uk/datasets/finn-b-CD2_NONHODGKIN_NAS_EXALLC/](https://gwas.mrcieu.ac.uk/datasets/finn-b-CD2_NONHODGKIN_NAS_EXALLC/) |
| Breast cancer | 2021 | 107722 | [https://gwas.mrcieu.ac.uk/datasets/finn-b-C3_BREAST_EXALLC/](https://gwas.mrcieu.ac.uk/datasets/finn-b-C3_BREAST_EXALLC/) |
| Alcohol consumption | 2017 | 112117 | [https://gwas.mrcieu.ac.uk/datasets/ieu-a-1283/](https://gwas.mrcieu.ac.uk/datasets/ieu-a-1283/) |
| Body mass index | 2022 | 99998 | [https://gwas.mrcieu.ac.uk/datasets/ieu-b-4816/](https://gwas.mrcieu.ac.uk/datasets/ieu-b-4816/) |
| Physical activity | 2022 | 78007 | [https://gwas.mrcieu.ac.uk/datasets/ieu-b-4860/](https://gwas.mrcieu.ac.uk/datasets/ieu-b-4860/) |
| Height | 2022 | 99997 | [https://gwas.mrcieu.ac.uk/datasets/ieu-b-4814/](https://gwas.mrcieu.ac.uk/datasets/ieu-b-4814/) |
| Smoking | 2021 | 138088 | [https://gwas.mrcieu.ac.uk/datasets/finn-b-SMOKING/](https://gwas.mrcieu.ac.uk/datasets/finn-b-SMOKING/) |
| Age when periods started (menarche) | 2018 | 243944 | [https://gwas.mrcieu.ac.uk/datasets/ukb-b-3768/](https://gwas.mrcieu.ac.uk/datasets/ukb-b-3768/) |
| Had menopause | 2018 | 211114 | [https://gwas.mrcieu.ac.uk/datasets/ukb-b-18105/](https://gwas.mrcieu.ac.uk/datasets/ukb-b-18105/) |
| Type 2 diabetes, strict (exclude DM1) | 2021 | 212351 | [https://gwas.mrcieu.ac.uk/datasets/finn-b-E4_DM2_STRICT/](https://gwas.mrcieu.ac.uk/datasets/finn-b-E4_DM2_STRICT/) |
| Illnesses of mother: Breast cancer | 2018 | 423458 | https://gwas.mrcieu.ac.uk/datasets/ukb-b-13584/ |
| Illnesses of siblings: Breast cancer | 2018 | 361809 | https://gwas.mrcieu.ac.uk/datasets/ukb-b-12227/ |

**Supplementary Table S2** Detailed information on GWAS data for Hodgkin lymphoma, non-Hodgkin lymphoma, and breast cancer obtained from UK-Biobank (grouped by different genders).

| **Phenotype** | **Participants** | **Source** |
| --- | --- | --- |
| Hodgkins lymphoma-bothsex | 361194 | https://broad-ukb-sumstats-us-east-1.s3.amazonaws.com/round2/additive-tsvs/20001_1052.gwas.imputed_v3.both_sexes.tsv.bgz |
| Hodgkins lymphoma-female | 194153 | https://broad-ukb-sumstats-us-east-1.s3.amazonaws.com/round2/additive-tsvs/20001_1052.gwas.imputed_v3.female.tsv.bgz |
| Non Hodgkin lymphoma-bothsex | 361141 | https://broad-ukb-sumstats-us-east-1.s3.amazonaws.com/round2/additive-tsvs/20001_1053.gwas.imputed_v3.both_sexes.tsv.bgz |
| Non Hodgkin lymphoma-female | 194153 | https://broad-ukb-sumstats-us-east-1.s3.amazonaws.com/round2/additive-tsvs/20001_1053.gwas.imputed_v3.female.tsv.bgz |
| Breast cacner-bothsex | 361194 | https://broad-ukb-sumstats-us-east-1.s3.amazonaws.com/round2/additive-tsvs/C_BREAST_3.gwas.imputed_v3.both_sexes.tsv.bgz |
| Breast cacner-female | 194174 | https://broad-ukb-sumstats-us-east-1.s3.amazonaws.com/round2/additive-tsvs/C_BREAST_3.gwas.imputed_v3.female.tsv.bgz |


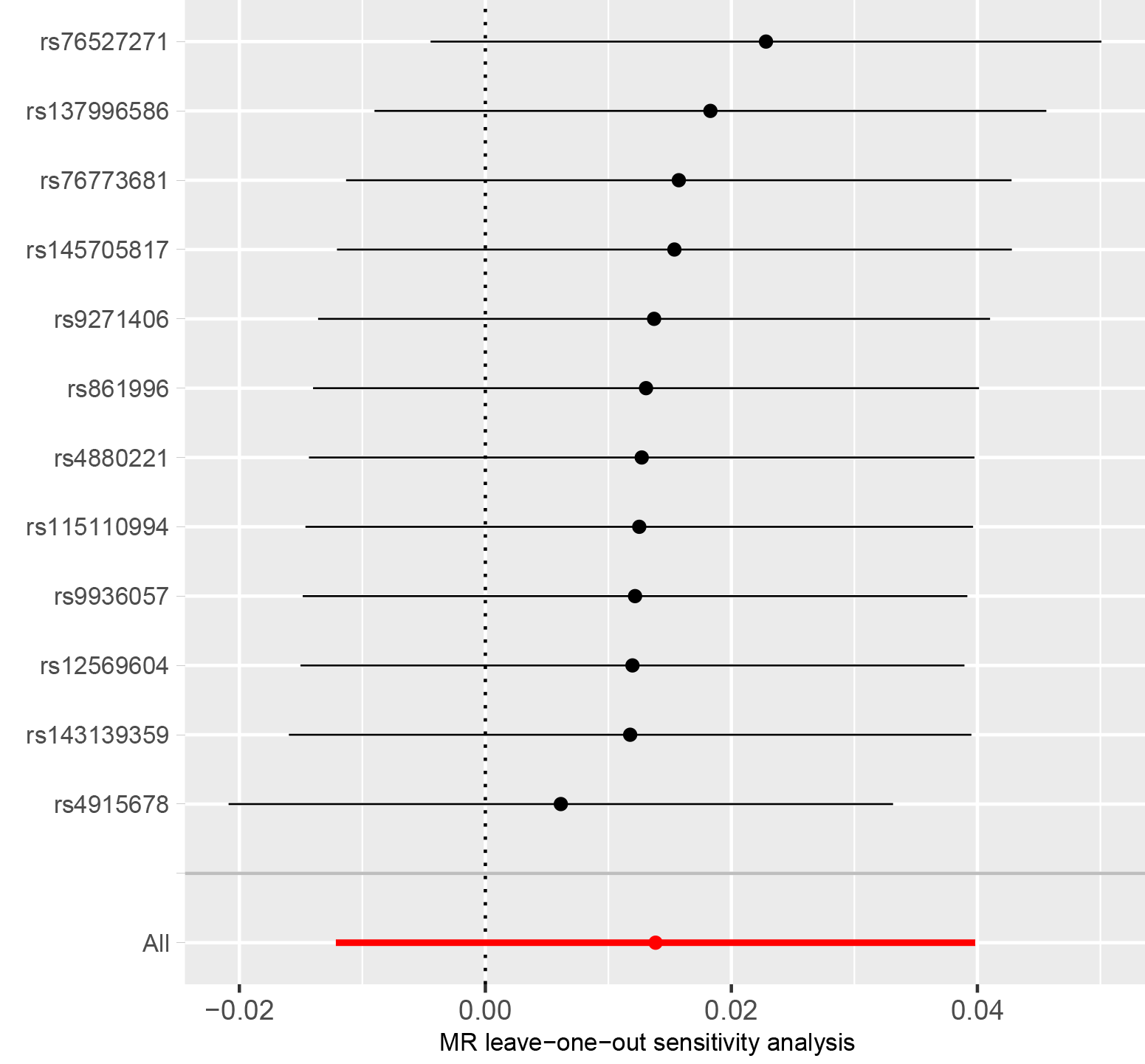


**Supplementary Figure S1** Leave-one-out method test of Hodgkin lymphoma and breast cancer risk (FinnGen consortium).


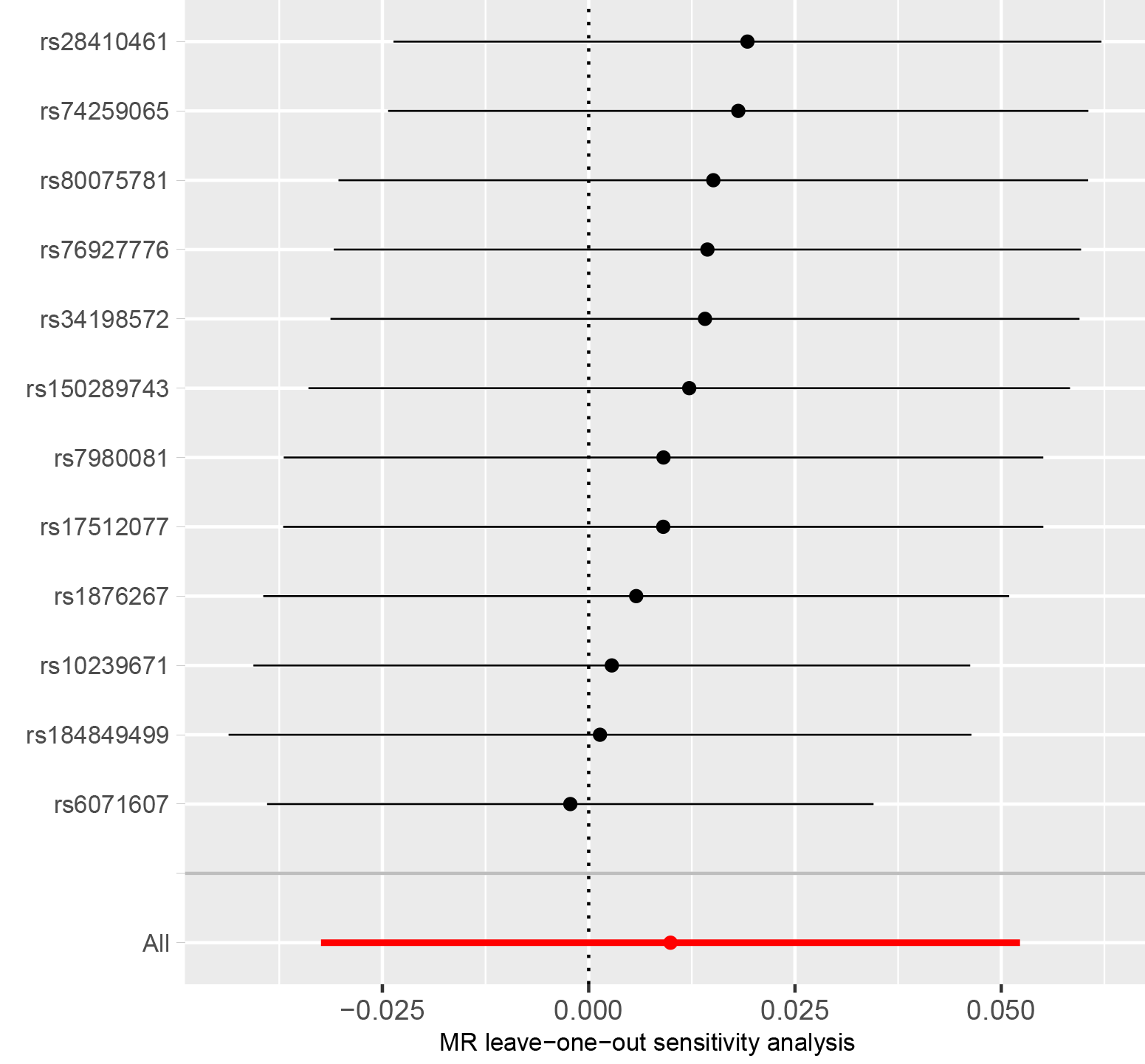


**Supplementary Figure S2** Leave-one-out method test of non Hodgkin lymphoma and breast cancer risk (FinnGen consortium).


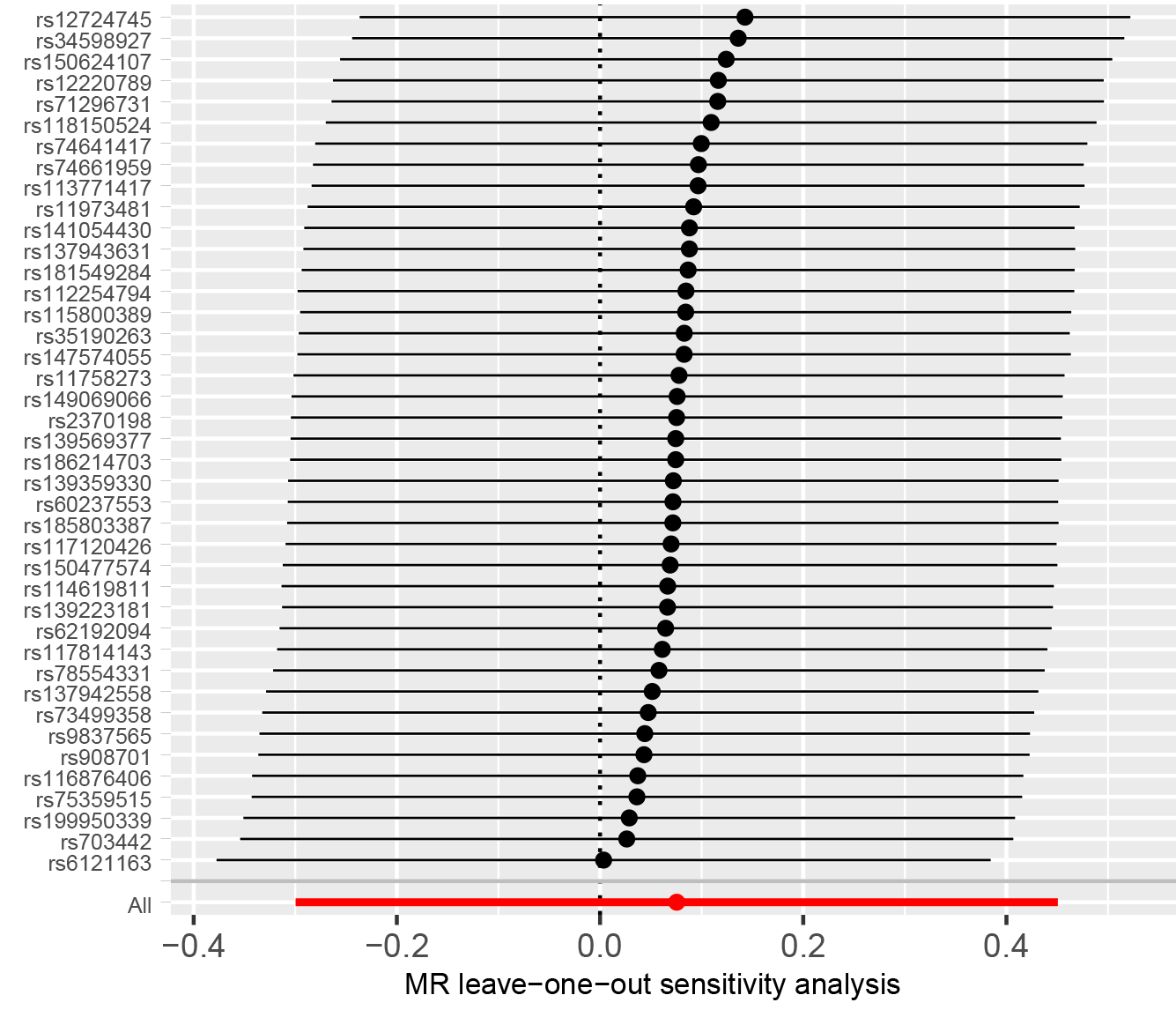


**Supplementary Figure S3** Leave-one-out method test of Hodgkin lymphoma with breast cancer risk for both genders (UK Biobank).


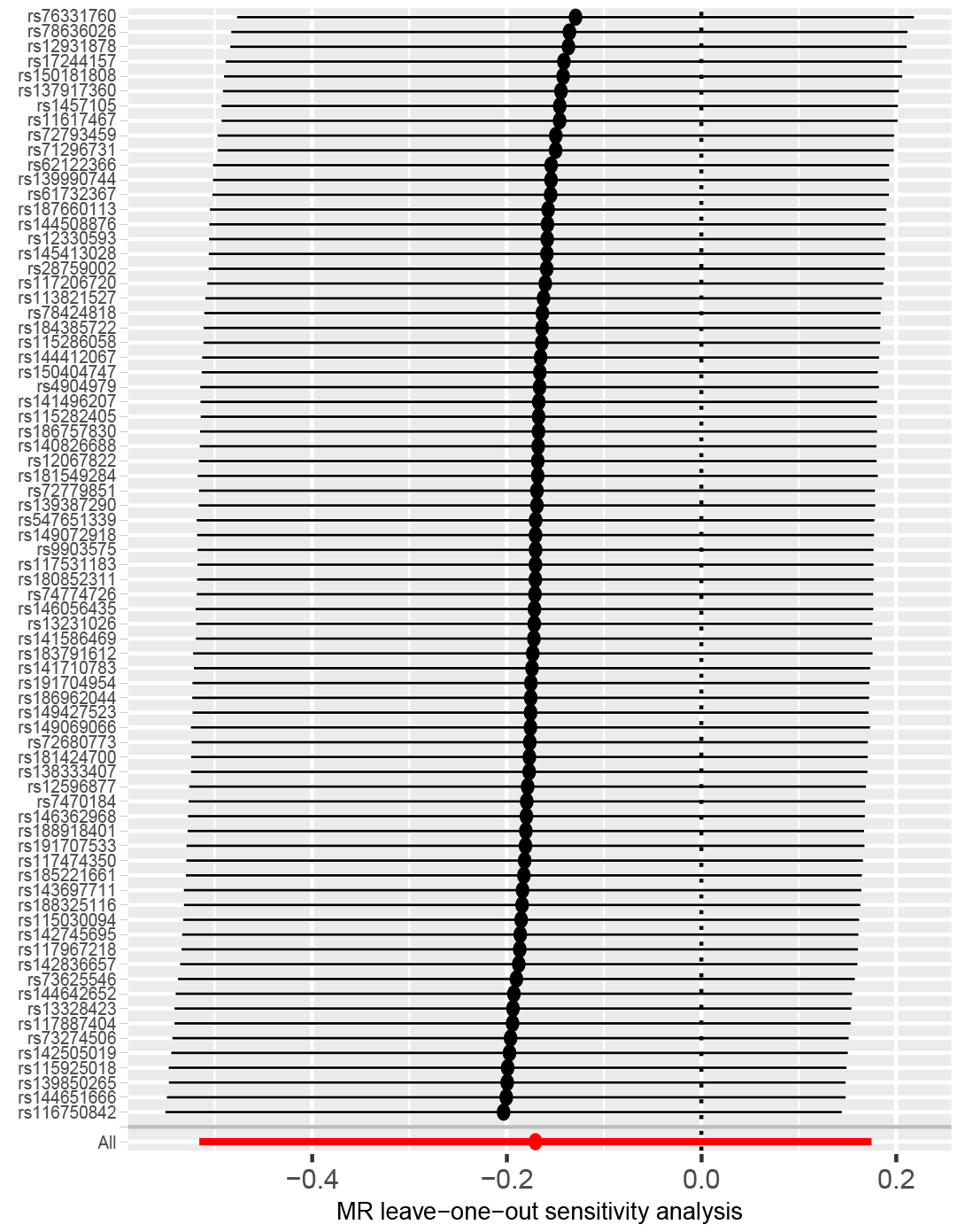


**Supplementary Figure S4** Leave-one-out method test of Hodgkin lymphoma with breast cancer risk for only female (UK Biobank).


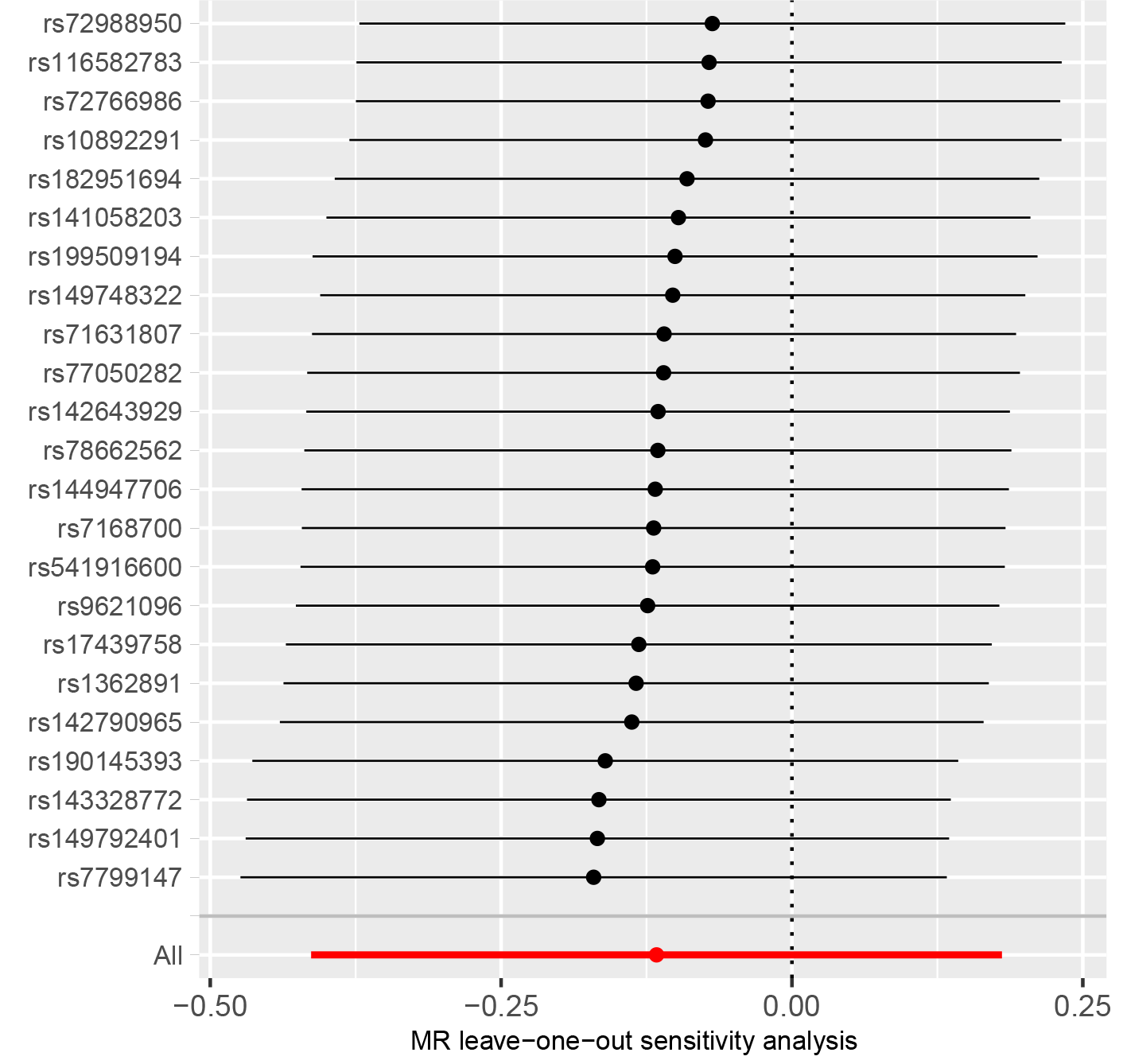


**Supplementary Figure S5** Leave-one-out method test of non Hodgkin lymphoma with breast cancer risk for both genders (UK Biobank).


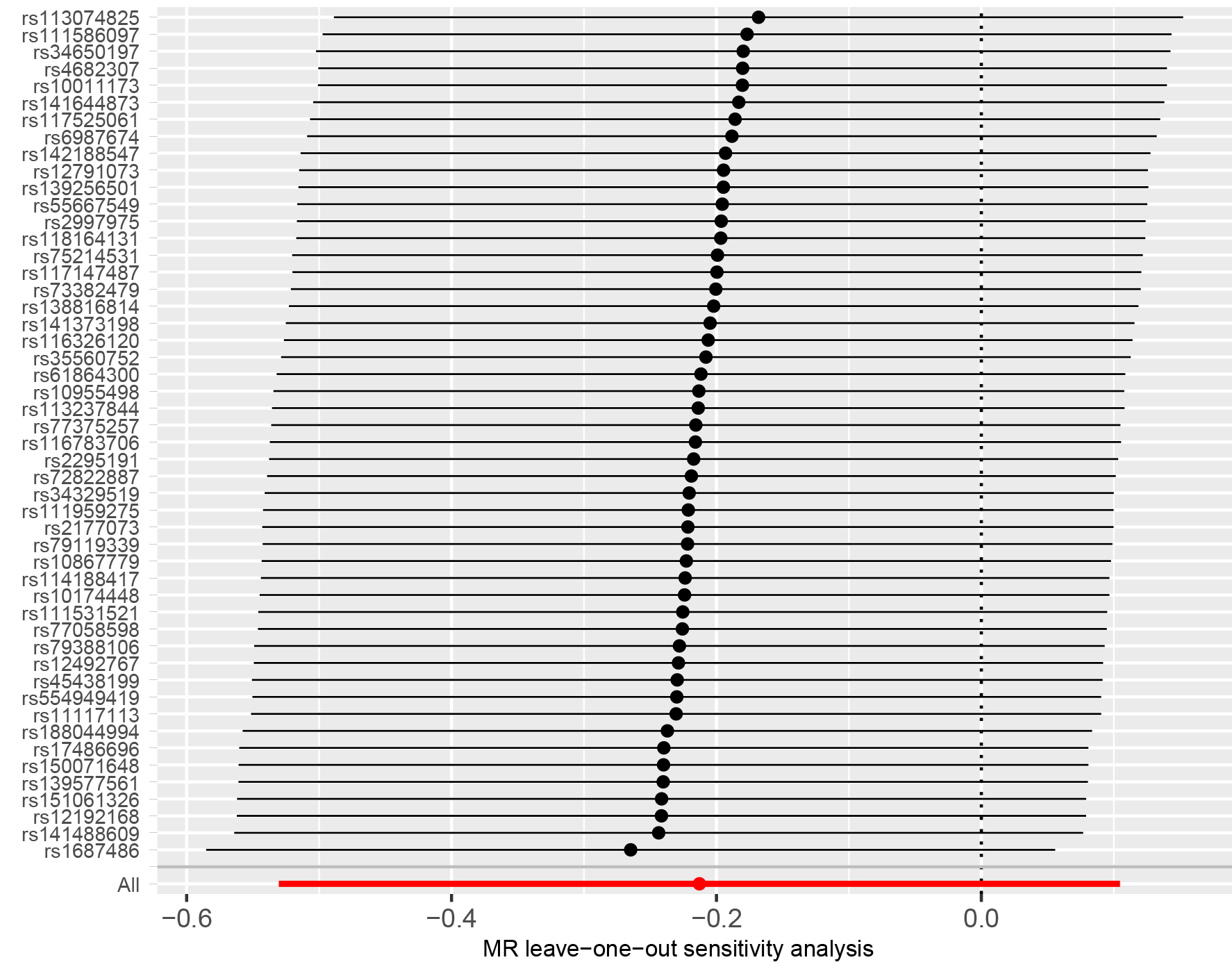


**Supplementary Figure S6** Leave-one-out method test of non Hodgkin lymphoma with breast cancer risk for only female (UK Biobank).


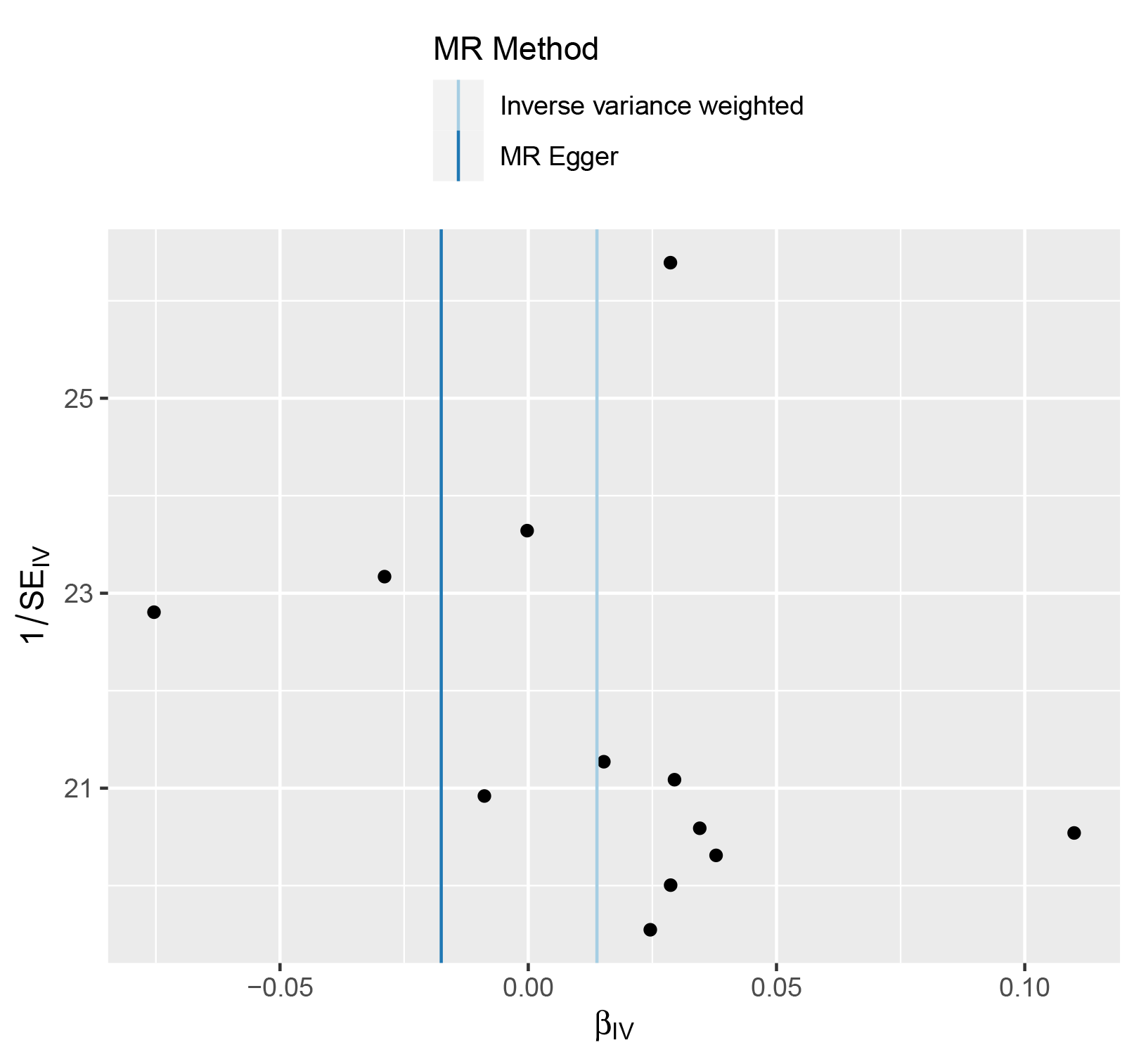


**Supplementary Figure S7** Funnel plot of Hodgkin lymphoma and breast cancer risk (FinnGen consortium).


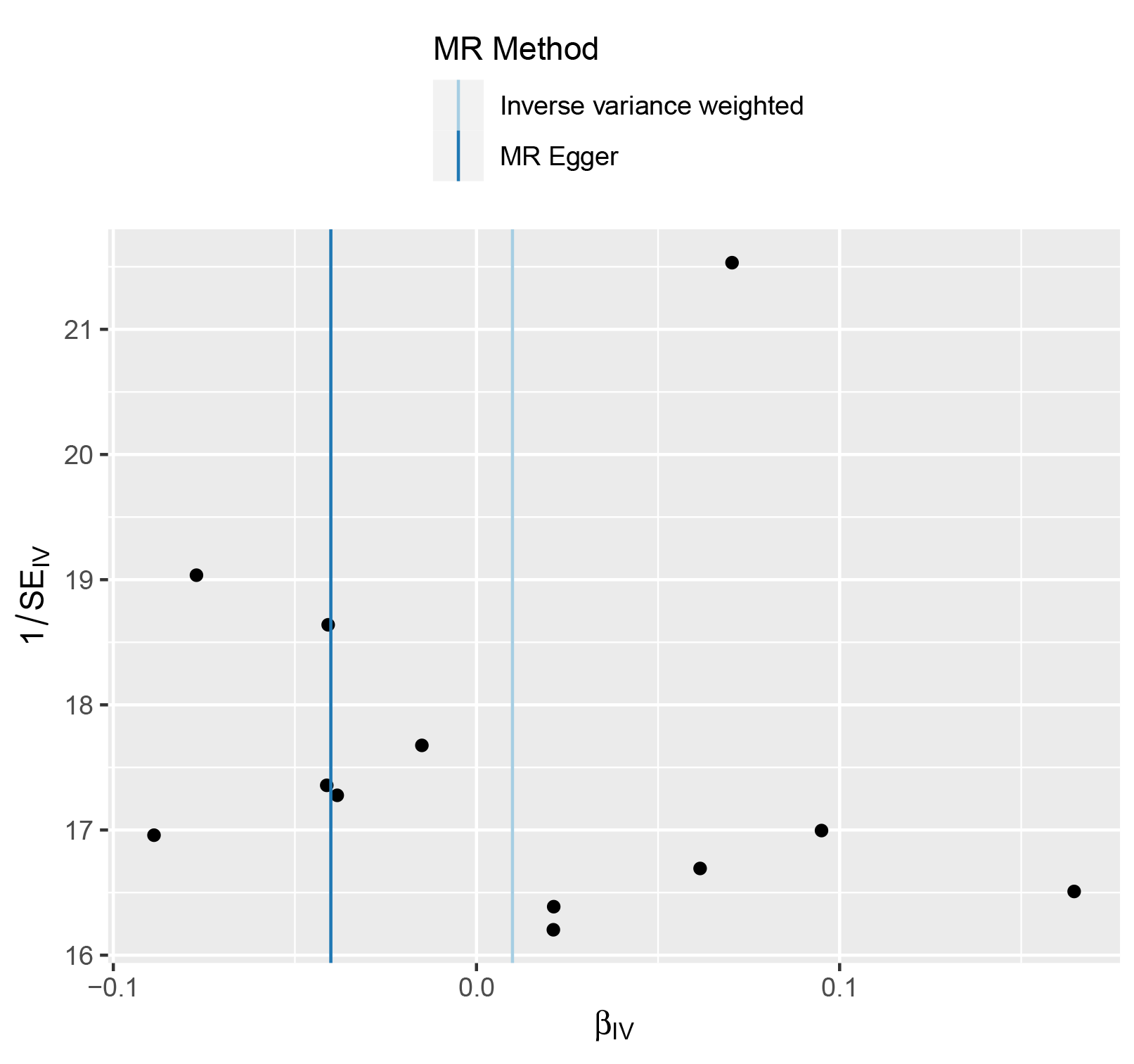


**Supplementary Figure S8** Funnel plot of non Hodgkin lymphoma and breast cancer risk (FinnGen consortium).


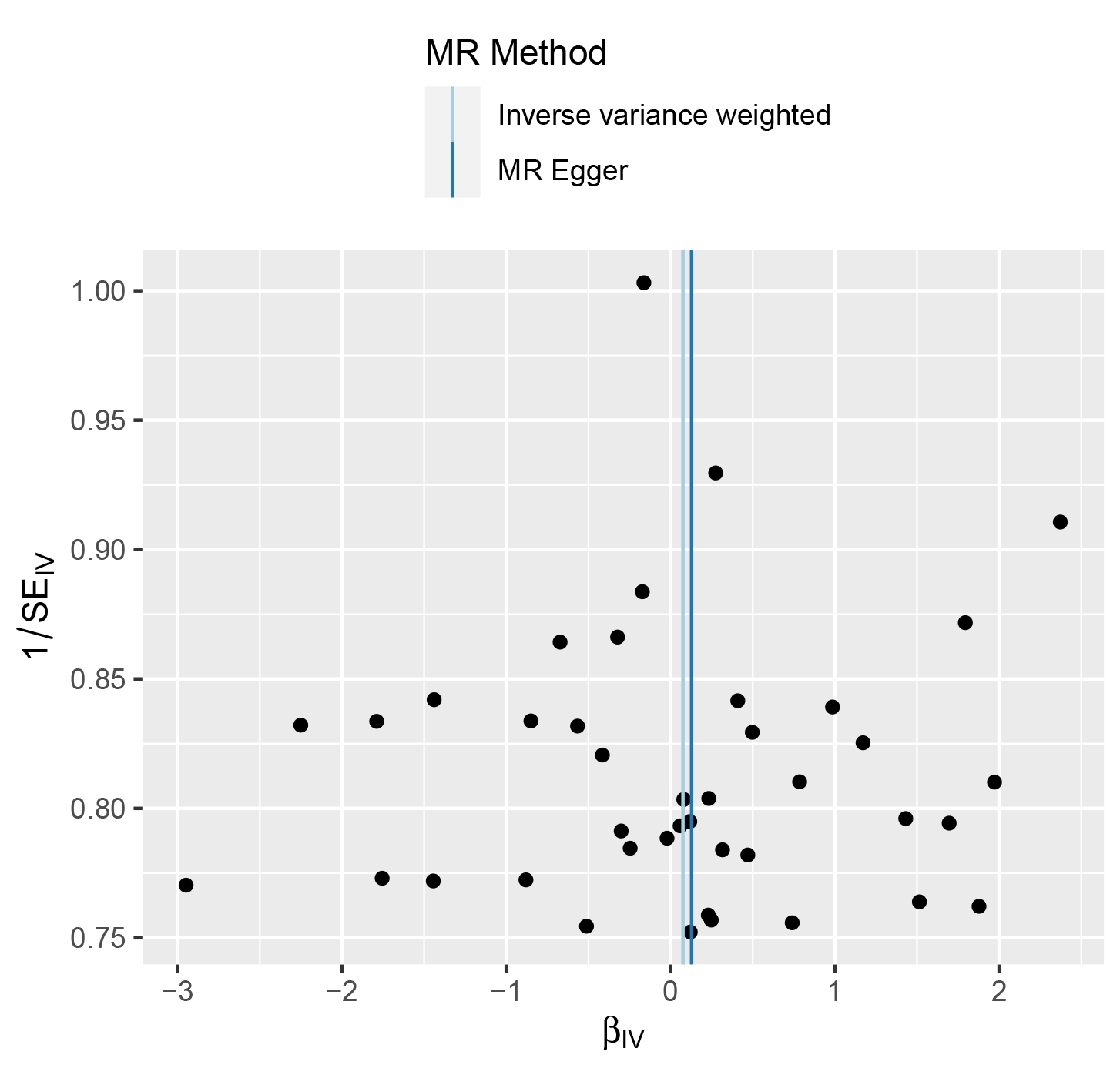


**Supplementary Figure S9** Funnel plot of Hodgkin lymphoma and breast cancer risk for both genders (UK Biobank).


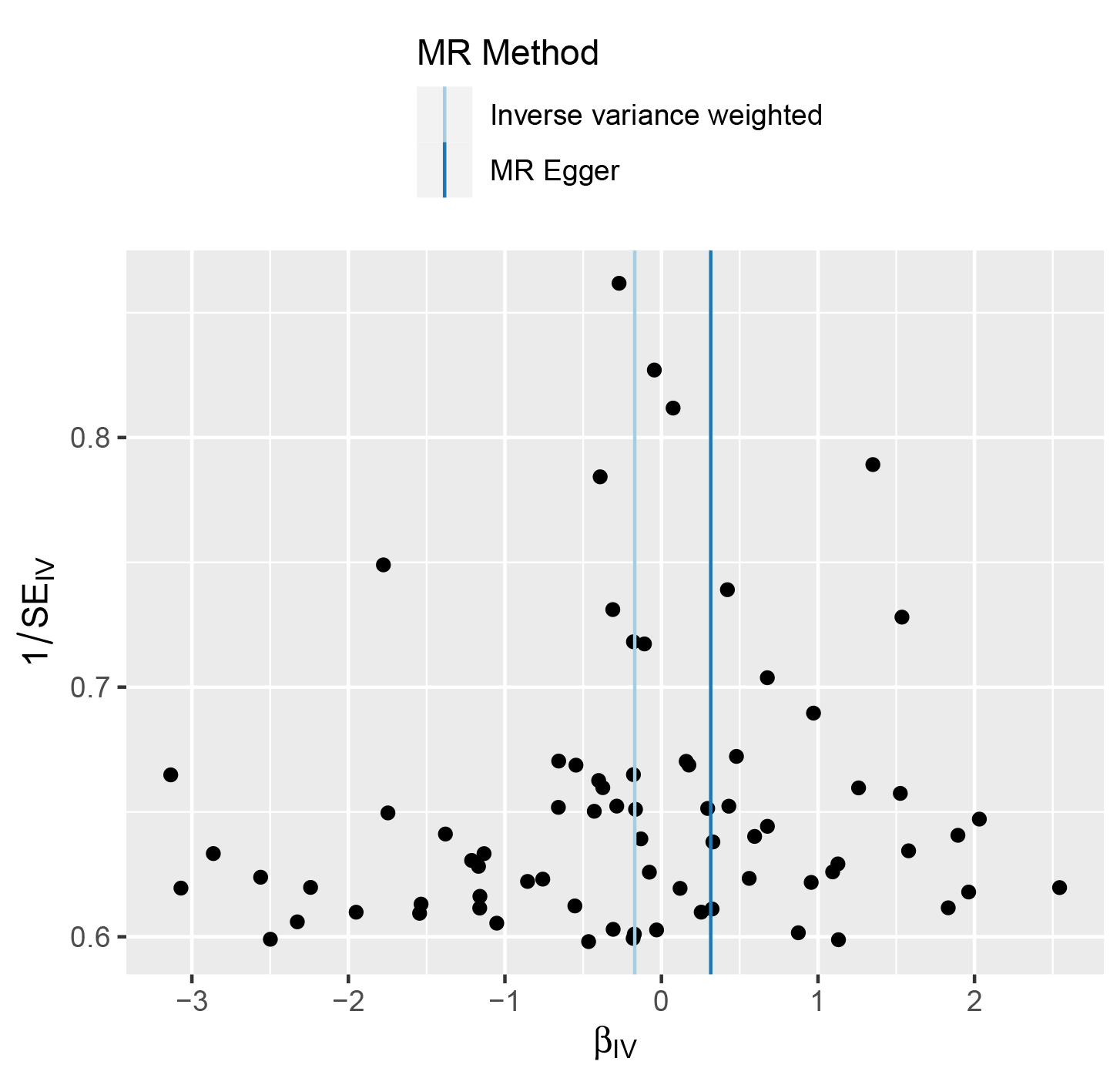


**Supplementary Figure S10** Funnel plot of Hodgkin lymphoma and breast cancer risk for only female (UK Biobank).


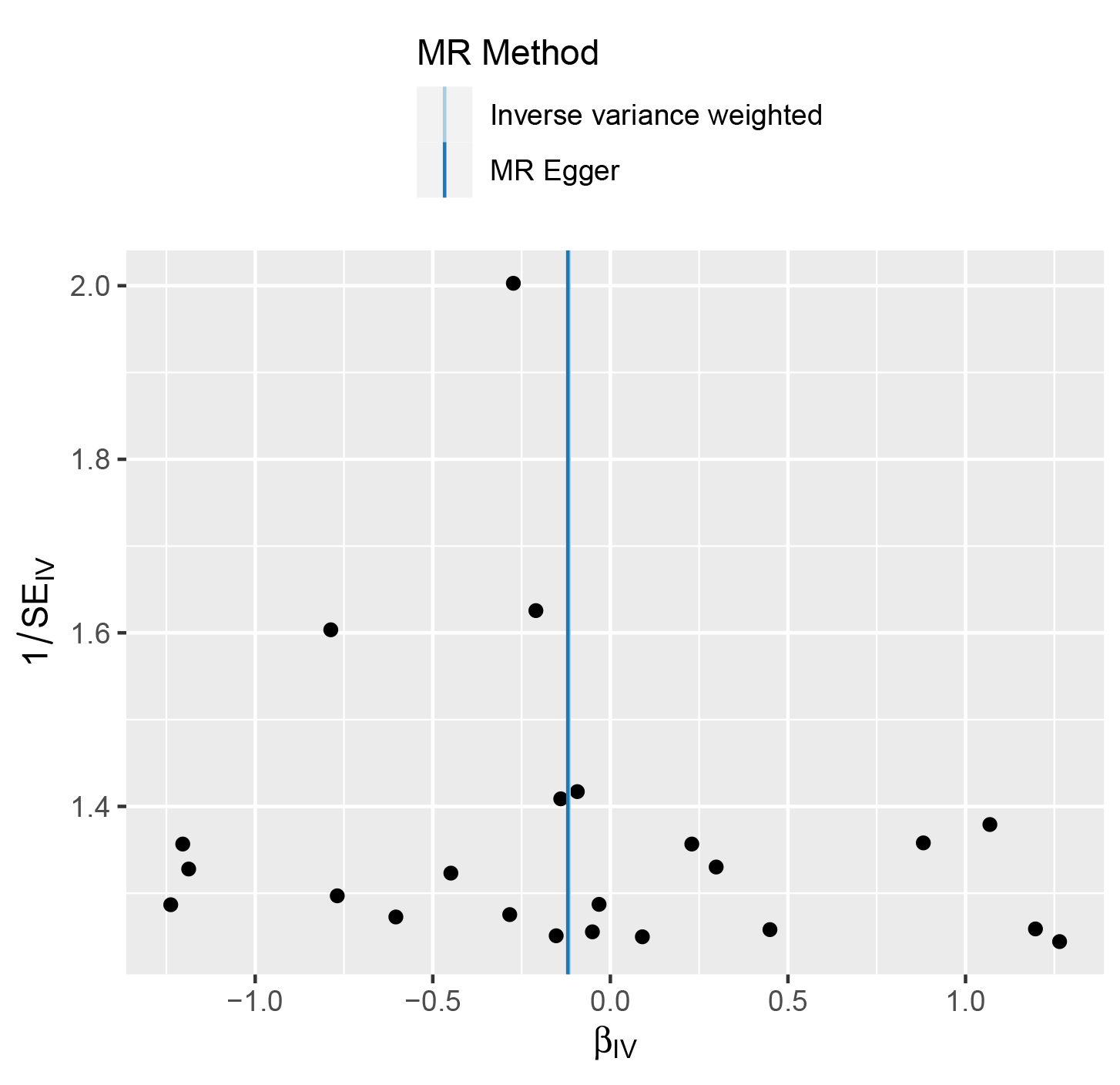


**Supplementary Figure S11** Funnel plot of non Hodgkin lymphoma and breast cancer risk for both genders (UK Biobank).


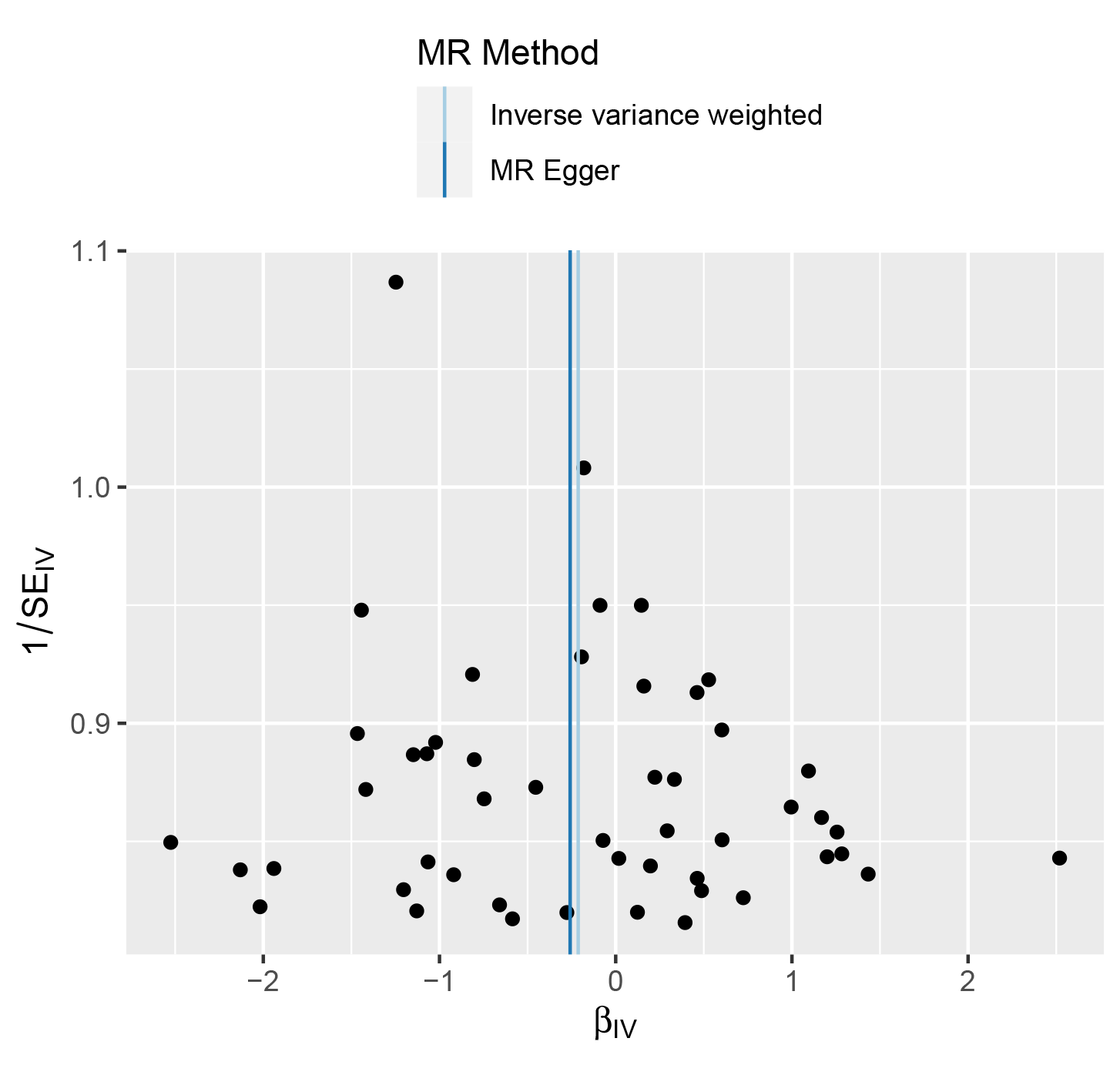


**Supplementary Figure S12** Funnel plot of non Hodgkin lymphoma and breast cancer risk for only female (UK Biobank).


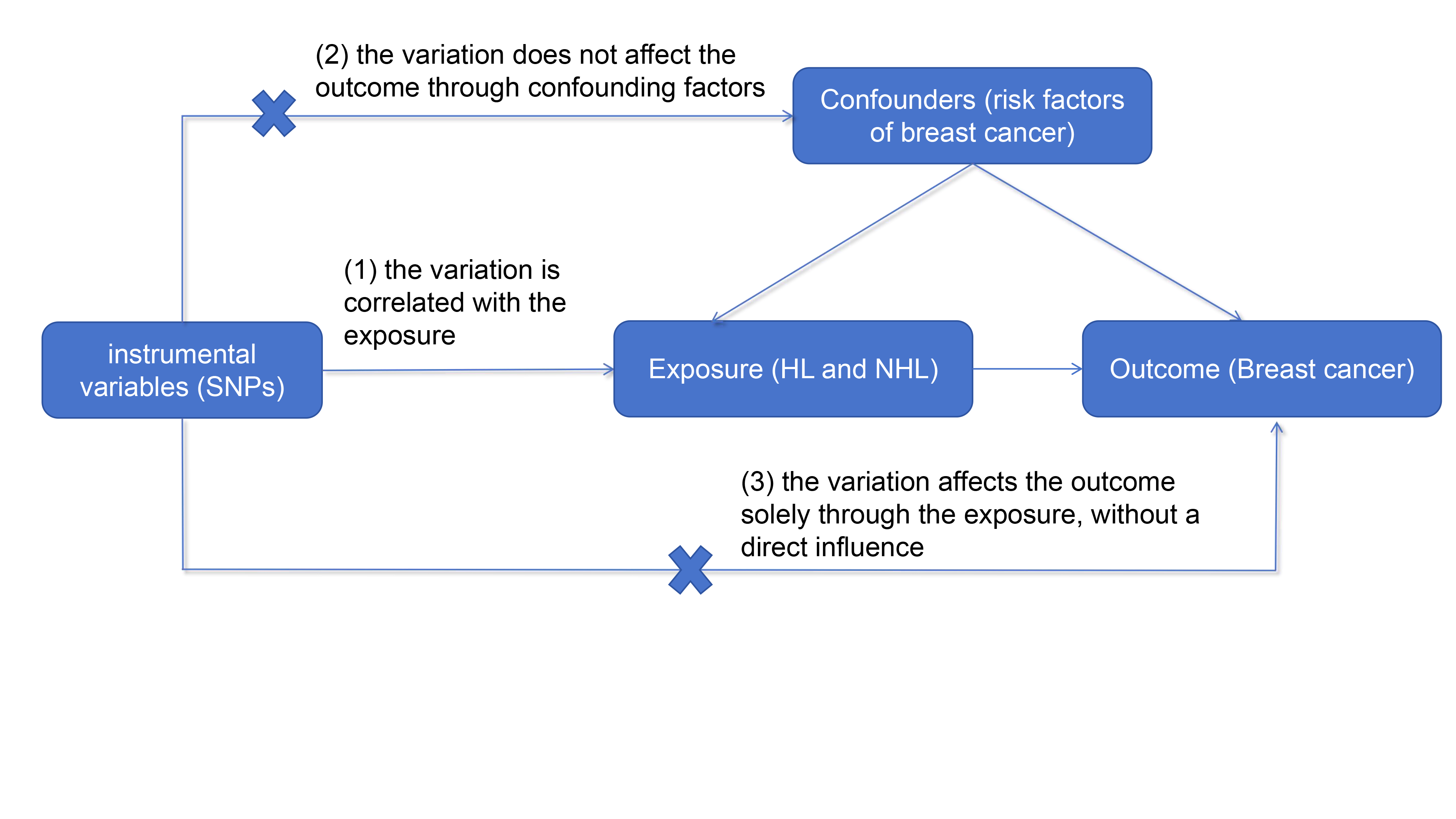


**Supplementary Figure S13** The genetic association between risk factors and outcomes.
